# Valentino’s Syndrome: A Life-Threatening Mimic of Acute Appendicitis

**DOI:** 10.5811/cpcem.2016.11.32571

**Published:** 2017-01-17

**Authors:** Christopher J. Amann, Andrea L. Austin, Sherri L. Rudinsky

**Affiliations:** Naval Medical Center San Diego, Department of Emergency Medicine, San Diego, California

## Abstract

Perforated ulcers are a rare cause of abdominal pain, and may not be considered when pain is localized to the right lower quadrant (RLQ). This case highlights an unusual presentation of a perforated duodenal ulcer that presented with RLQ pain, which has been described as Valentino’s syndrome. Valentino’s syndrome occurs when gastric or duodenal fluids collect in the right paracolic gutter causing focal peritonitis and RLQ pain. This case highlights that perforated ulcers, while an uncommon cause of RLQ pain, must remain on the differential of any patient that has an abdominal examination consistent with peritonitis.

## INTRODUCTION

Approximately six million people in the United States have peptic ulcer disease (PUD) with an annual direct cost estimate of $3.1 billion.^1^ *Helicobacter pylori* infection and nonsteroidal anti-inflammatory drug (NSAID) use are the two main causes. With improved understanding of the pathogenesis of PUD and treatment with antibiotics, proton pump inhibitors (PPIs), avoidance of NSAIDs and advanced endoscopy techniques, the incidence of complications from PUD requiring surgical intervention has decreased to approximately 11%.^2^ While a rare event, ulcer perforation is associated with high mortality at 10.6%, and emergency physicians (EP) must remain vigilant.^2^ This case highlights Valentino’s syndrome, in which the patient presents with pain in the right lower quadrant (RLQ) of the abdomen due to perforation of a duodenal ulcer through the retroperitoneum.^3^,^4^,^5^,^6^ To our knowledge, this is only the second reported case of Valentino’s syndrome in the emergency medicine literature.

## CASE REPORT

An 18-year-old woman presented to the ED with one-day history of RLQ abdominal pain. She described the pain as constant with an acute worsening approximately three hours prior to arrival. The pain was sharp, 10/10 in intensity, and associated with diaphoresis, nausea, vomiting, and anorexia. She reported taking ibuprofen for her symptoms, which provided little relief. She denied a history of dysuria, change in vaginal discharge, or being sexually active. She was seen by her primary care physician 12 days prior with similar but less severe pain. She was diagnosed with gastritis and prescribed ranitidine 150mg twice daily and ondansetron 4mg as needed for her symptoms. She was otherwise healthy, with no reported prior medical problems or surgeries. She was a senior in high school, lived with her parents and did not smoke, drink alcohol or use illicit substances.

On physical exam the patient’s vital signs were temperature 98.0°F, pulse 85, blood pressure 128/72, respiratory rate 18, and pulse oximetry 98% on room air. She was well nourished but in obvious distress from pain. Abdominal examination revealed tenderness over McBurney’s point with focal peritonitis. Pelvic exam was unremarkable, with normal discharge and without cervical motion tenderness or adnexal masses or tenderness. Rectal examination was performed, which demonstrated brown Guaiac negative stool.

Laboratory data were remarkable for a negative urine HCG. Urinalysis showed small leukocyte esterase, nitrite negative and without hematuria or pyuria on microscopic analysis. Her white blood cell count was 6,200 mcl, otherwise normal complete blood count, electrolytes and creatinine. Serum lactate was 0.96 mEq/L. Liver function tests showed an elevated LDH of 519 U/L, normal bilirubin and transaminases. Lipase was elevated at 87 U/L.

**Image 1:**
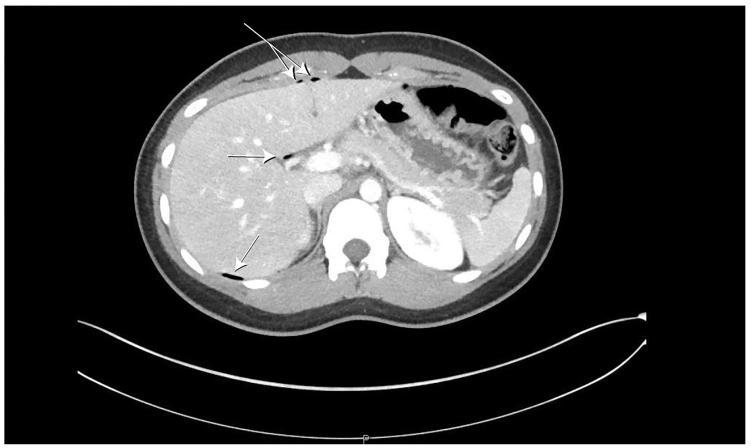
Axial CT image demonstrating free air (arrows) in superior abdomen.

**Image 2:**
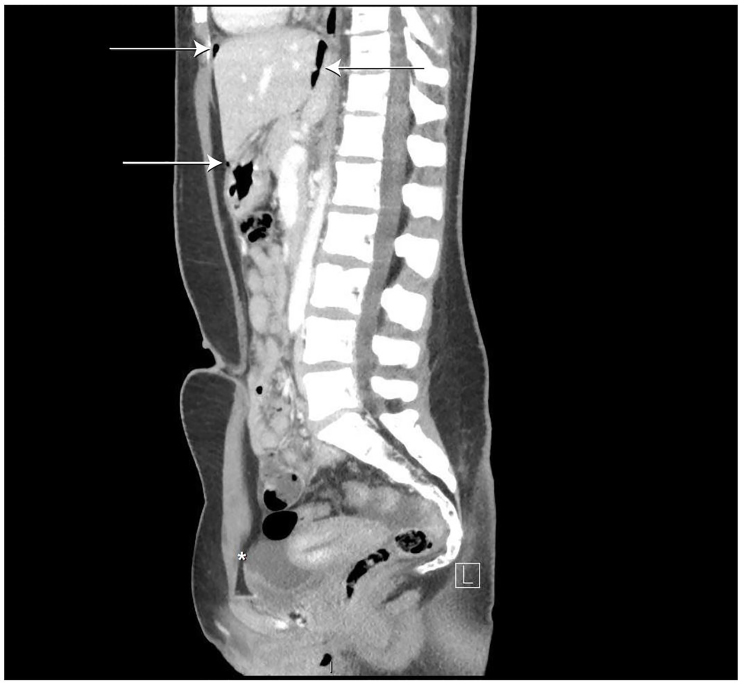
Sagittal CT image demonstrating free air (arrows) and free fluid (asterisk) in abdomen.

The time course of symptoms and the degree of distress initially led the EP to consider ovarian torsion, but with the tenderness localizing to McBurney’s point and normal pelvic examination, a computed tomography (CT) of the abdomen pelvis was ordered. The study was protocoled for appendicitis (intravenous contrast only). This study revealed pneumoperitoneum centered within the greater sac of the upper anterior abdomen and free fluid in the pelvis (Images 1 and 2). This was favored to be secondary to a perforated duodenal ulcer and general surgery was emergently consulted.

The patient was admitted to the general surgery service and underwent an exploratory laparoscopy. She was found to have a small, perforated ulcer in the first portion of her duodenum, which was repaired with a tongue of omentum, known as a Graham patch.^7^ Her postoperative course was uneventful. Serologic testing for *H. pylori* antibody was significantly elevated at 2.8, U/ml, indicating an active infection, and her gastrin level was normal. She was treated for *H. pylori* with amoxicillin/clarithromycin/bismuth and was discharged on postoperative day 3. Four weeks later, upon completion of triple therapy, upper endoscopy demonstrated chronic gastritis and biopsies were negative for *H. pylori*.

## DISCUSSION

Valentino’s syndrome occurs when gastric or duodenal fluid collects in the right paracolic gutter causing focal peritonitis and RLQ pain.^3^,^4^,^5^,^6^ The syndrome is named after the 1920s silent film star Rudolph Valentino. In 1926, Valentino collapsed in a New York City hotel and underwent surgery for presumed appendicitis at New York Polytechnic Hospital. At the time he was found to have a perforated ulcer. Postoperatively, he developed peritonitis, multiple organ system dysfunction and died several days later.^8^ His case gained significant notoriety due to his fame at the time, and has since become a cautionary tale to medical students and residents.

Gastric and duodenal ulcers are often collectively referred to as PUD because of the similarity in their pathogenesis and treatment. *H. pylori* and NSAIDs contribute the most to PUD. Complications from PUD include hemorrhage, obstruction, cancer, and perforation. A better understanding of the risk factors for PUD has led to a significant decrease in complications of the disease. Total admissions for PUD have decreased by almost 30% since the 1990s. The percentage of patients who require emergent surgery for complicated disease has decreased, to approximately 11%. Perforation has the highest mortality rate of any complication of ulcer disease, approaching 15%. Despite a decrease in reported ulcer-related mortality, from 3.9% in 1993 to 2.7% in 2006, over 4,000 estimated deaths are caused by PUD each year.^2^

Perforation of an anterior duodenal ulcer allows for free communication of duodenal and gastric contents into the peritoneal cavity. These contents will collect in dependent portions of the peritoneum, which is often the RLQ.^3^ If the patient seeks medical attention early in the course of the disease, he or she may have poorly localized pain. Localized pain to the RLQ can mimic acute appendicitis so closely that surgical exploration without imaging has lead to the diagnosis being made intra-operatively.^3^ As time passes, this can progress to focal tenderness, as in this case, or to generalized peritonitis. Initial imaging other than CT may demonstrate free fluid around a normal appendix on ultrasound and free air around the kidney, or “veiled kidney sign” on abdominal radiographs.^6^ This patient was hemodynamically stable with focal peritonitis consistent with a surgical abdomen with the diagnosis made on CT imaging.

Definitive treatment for a perforated duodenal ulcer is surgical. This patient underwent a laparoscopic Graham patch repair and had an uneventful postoperative course. According to primary care notes, she reported several weeks of abdominal pain, likely a result of gastritis/PUD, presumably from her *H. pylori* infection. Treatment with NSAIDs may have exacerbated her disease, leading to her complication of perforation. Given her age, Zollinger-Ellison syndrome – a gastrin-secreting tumor, most commonly found in the exocrine pancreas – was also on the differential. However, a serum gastrin level was obtained which was normal, effectively ruling out this disease.

## CONCLUSION

While rare, especially in young people, perforated gastric and duodenal ulcers have a high morbidity and mortality. Air and liquid from perforated viscous can track to various locations in the abdomen. Thus, in any patient with a peritoneal exam, regardless of the location of this pain, perforated ulcer should remain on the differential.
